# In Vitro Evaluation of Esters of Quinoxaline-1,4-di-*N-*oxide Derivatives as New Antitaeniasis Agents and Their Inhibitory Activity Against Triosephosphate Isomerase

**DOI:** 10.3390/ph18030406

**Published:** 2025-03-13

**Authors:** Francisca Palomares-Alonso, Alonzo González-González, Alma D. Paz-González, Eyra Ortiz-Pérez, Ana Verónica Martínez-Vázquez, Itzhel García-Torres, Gabriel López-Velázquez, Helgi Jung-Cook, Gildardo Rivera

**Affiliations:** 1 Laboratorio Para el Estudio de la Neuro-Inflamación, Instituto Nacional de Neurología y Neurocirugía, Ciudad de México 14269, Mexico; ingpalomares@yahoo.com.mx; 2 Laboratorio de Biotecnología Farmacéutica, Centro de Biotecnología Genómica, Instituto Politécnico Nacional, Reynosa 88710, Mexico; al.gonzalez.gonzalez88@gmail.com (A.G.-G.); apazg@ipn.mx (A.D.P.-G.); eortizp@ipn.mx (E.O.-P.); avmartinez@ipn.mx (A.V.M.-V.); 3 Laboratorio de Biomoléculas y Salud Infantil, Instituto Nacional de Pediatría, México City 04530, Mexico; garciaitzhel@gmail.com (I.G.-T.); glv_1999@ciencias.unam.mx (G.L.-V.); 4 Departamento de Farmacia, Facultad de Química, Universidad Nacional Autónoma de México, Ciudad de México 04510, Mexico; helgi@unam.mx

**Keywords:** *Taenia solium*, quinoxaline-1,4-di-*N*-oxide, triosephosphate isomerase, molecular docking

## Abstract

**Background/Objectives:** Pork tapeworm *Taenia solium* is the causative agent of cysticercosis which may develop in muscle tissue, skin, eyes, and the central nervous system (neurocysticercosis). It is estimated by the World Health Organization (WHO) that about 2.56–8.30 million are infected worldwide. Praziquantel and albendazole are used for anthelminthic treatment of neurocysticercosis; however, not all patients have a complete elimination of cysts, which makes it necessary to seek new and improved treatment options. **Methods:** In this study, methyl, ethyl, n-propyl, and iso-propyl quinoxaline-7-carboxylate-1,4-di-*N*-oxide derivatives were evaluated in vitro against *Taenia crassiceps (T. crassiceps)* cysts. Additionally, to know their potential mode of action, a molecular docking analysis on *T. solium* triosephosphate isomerase (*Ts*TIM) and an enzyme inactivation assay on recombinant *Ts*TIM were carried out. **Results:** Nine compounds had time- and concentration-dependent cysticidal activity. Particularly, compounds TS-12, TS-19, and TS-20 (EC_50_ values 0.58, 1.02, and 0.80 µM, respectively) were equipotent to albendazole sulfoxide (EC_50_ = 0.68 µM). However, TS-12 compounds only cause a slight inhibition of *Ts*TIM (<40% at 1000 µM), suggested that another drug target is implicated in the biological effects. **Conclusions:** These results demonstrated that quinoxaline 1,4-di-*N*-oxide is a scaffold to develop new and more potent antitaeniasis agents, although it is necessary to explore other pharmacological targets to understand their mode of action.

## 1. Introduction

Pork tapeworm *Taenia solium* is the causative agent for two infections in humans, taeniasis (intestinal infection caused by adult tapeworms) and cysticercosis (caused by the larvae form), which could develop in the muscles, skin, eyes, and central nervous system (CNS). The ingested *T. solium* eggs commonly hatch in the gastrointestinal (GI) tract; once in the larval stage, they migrate through tissues, where these larvae may form cysts in muscle tissue and cause subcutaneal lumps. Similarly, they can lodge nerve tissue, among the most important, in the brain and the spinal cord. When the cysts are in nerve tissue, this often results in hydrocephalus, motor deficits, seizures, and in the most severe cases, even death. A less typical tissue for *T. solium* cyst formation is in the eyes; in such cases, it may result in visual changes and, in some cases, complete blindness [1,2,3]. Normally, cysticercosis is asymptomatic; however, some symptoms include severe headaches, blindness, convulsions, and epileptic seizures. In the case of epilepsy, it is estimated that about 30% of epilepsy worldwide is caused by neurocysticercosis in areas where the parasite is endemic [2,4,5,6]. The endemic areas include most of Latin America, Asia, and Africa since the parasite life cycle is related to an environment where there are free-ranging pigs and often open defecation conditions, such is the case in impoverished areas common to these parts of the world [7,8,9]. The World Health Organization (WHO) estimates that about 2.56–8.30 million are infected worldwide, including symptomatic and asymptomatic cases [8].

Taeniasis is easily eliminated with anthelmintic drugs; however, the treatment for cysticercosis is complex, as the cyst’s destruction may elicit an inflammatory response. While the cysticercus is alive, it triggers an immunomodulatory reaction that suppresses the inflammatory response, allowing the parasite to survive [10]. However, when the cysticercus dies, this immunomodulation ceases, leading to an inflammatory response [11]. Thus, this type of infection requires specialized treatment, including long courses with high doses of praziquantel and/or albendazole, antiepileptic treatment [8], and corticosteroids to prevent severe inflammation [11]. Still, there are failed treatments under this anthelminthic regime [12] that make it necessary to continue searching for additional anthelminthic alternatives. The treatment should act specifically toward the parasite, such that the host may not deal with further secondary effects additional to the inflammatory response.

**Figure 1:**
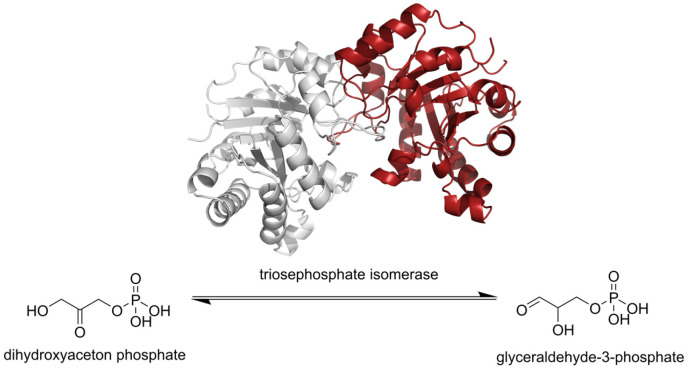
Diagram for the activity carried out by the target triosephosphate isomerase.

*T. solium* is a helminth that depends on the glycolysis pathway for energy production, and several central metabolic enzymes from these parasites have been tested and considered as candidates for drug design [13]. Such is the case for triosephosphate isomerase (TIM), which has been widely studied as a target for rational drug design in protozoan parasites [14,15,16,17,18,19,20]. The relevance of TIM as a pharmacological target resides in that it catalyzes the interconversion of the dihydroxyacetone phosphate and glyceraldehyde-3-phosphate in the fifth step of the glycolytic pathway (Figure 1).

Now, as for the chemical scaffold of interest, quinoxaline-1,4-di-*N*-oxide (QNO) has been successfully studied for the development of new bactericidal, antitumoral, fungicidal, trichomonacidal, trypanocidal, and leishmanicidal agents [21,22,23,24,25,26,27,28,29,30,31]. Interestingly, quinoxaline-1,4-di-*N*-oxide had antiparasitic activity at the micromolar level against cestode and nematode (*Equinococcus granulosus* and *Fasciola hepatica*) [32]. Therefore, it is interesting to explore the anthelmintic effect that quinoxaline-1,4-di-*N*-oxide derivatives have against *Taenia* cysts.

In the last few years, quinoxaline-1,4-di-*N*-oxide derivatives have emerged as TIM inhibitors against different parasites; for example, our research group reported the inhibition of *Trichomona vaginalis* TIM (*Tv*TIM) by a 7-methyl ester quinoxaline-1,4-di-*N*-oxide derivative and the inhibition of *Giardia lamblia* TIM (*Gl*TIM) by an isobutyl ester quinoxaline-1,4-di-*N*-oxide derivative, which is potent and selective [33]. Therefore, in this work, we propose the in vitro analysis of a series of esters of quinoxaline-1,4-di-*N*-oxide derivatives against *T. crassiceps* cysticercus and their in silico and in vitro evaluation on *T. solium* TIM (*Ts*TIM) to determine the mode of action. To our knowledge, this is the first evidence that will support the development of new antitaenia agents using the quinoxaline-1,4-di-*N*-oxide scaffold.

## 2. Results

### 2.1. Antitaenia Activity

The quinoxaline-1,4-di-*N*–oxide derivatives were evaluated up to a maximum of 5 µg/mL (equivalent to a molarity ranging from 10.82–17.23 µM) to determine their antitaeniasis activity (Table 1). Quinoxaline-1,4-di-*N*–oxide derivatives had EC_50_ values from 0.58 to 17.23 µM, and the reference drug albendazole sulfoxide had an EC_50_ value of 0.68 µM. Three compounds (TS-12, TS-19, and TS-20) had EC_50_ values equal or similar to the reference drug.

### 2.2. Molecular Docking

**Figure 2:**
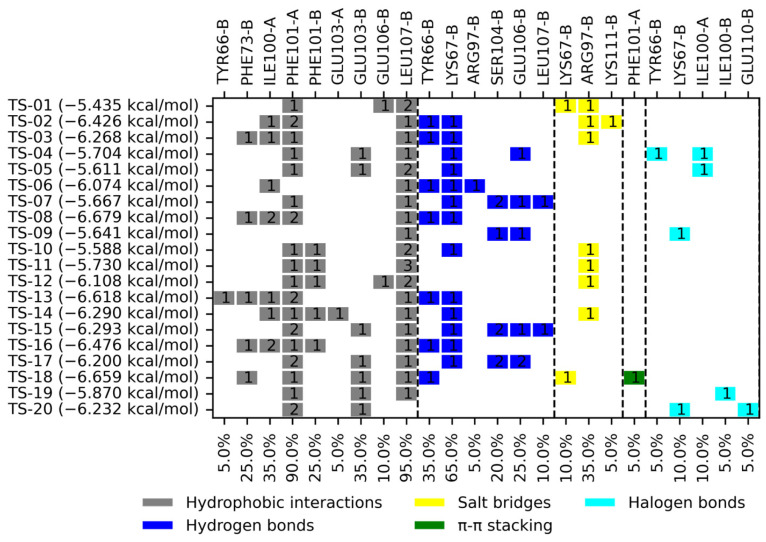
Summary of binding free energies and interaction profiles for quinoxaline 1,4-di-*N*-oxide derivatives on *Ts*TIM. The number within the box reflects the number of interactions of that type for a given residue.

**Figure 3:**
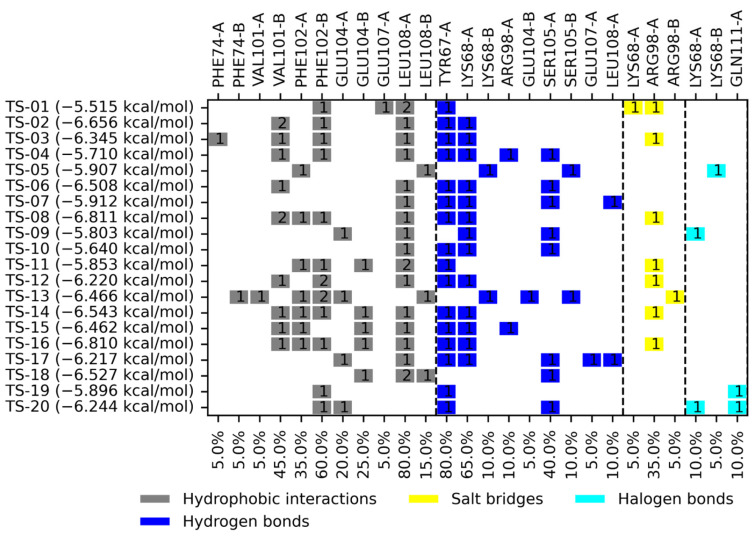
Summary of binding free energies and interaction profiles for quinoxaline 1,4-di-*N*-oxide derivatives on *Hs*TIM. The number within the box reflects the number of interactions of that type for a given residue.

A molecular docking analysis was carried out on *Ts*TIM and *Hs*TIM, considering binding free energy (BFE) values and interactions profile to propose compounds with the most potential to act as *Ts*TIM inhibitors in a selective manner. Figure 2 and Figure 3 summarize the results obtained. The BFE values for the quinoxaline 1,4-di-*N*-oxide derivatives on *Ts*TIM range from −5.435 to −6.679 kcal/mol.

### 2.3. Enzymatic Activity Evaluation Against TsTIM

**Figure 4:**
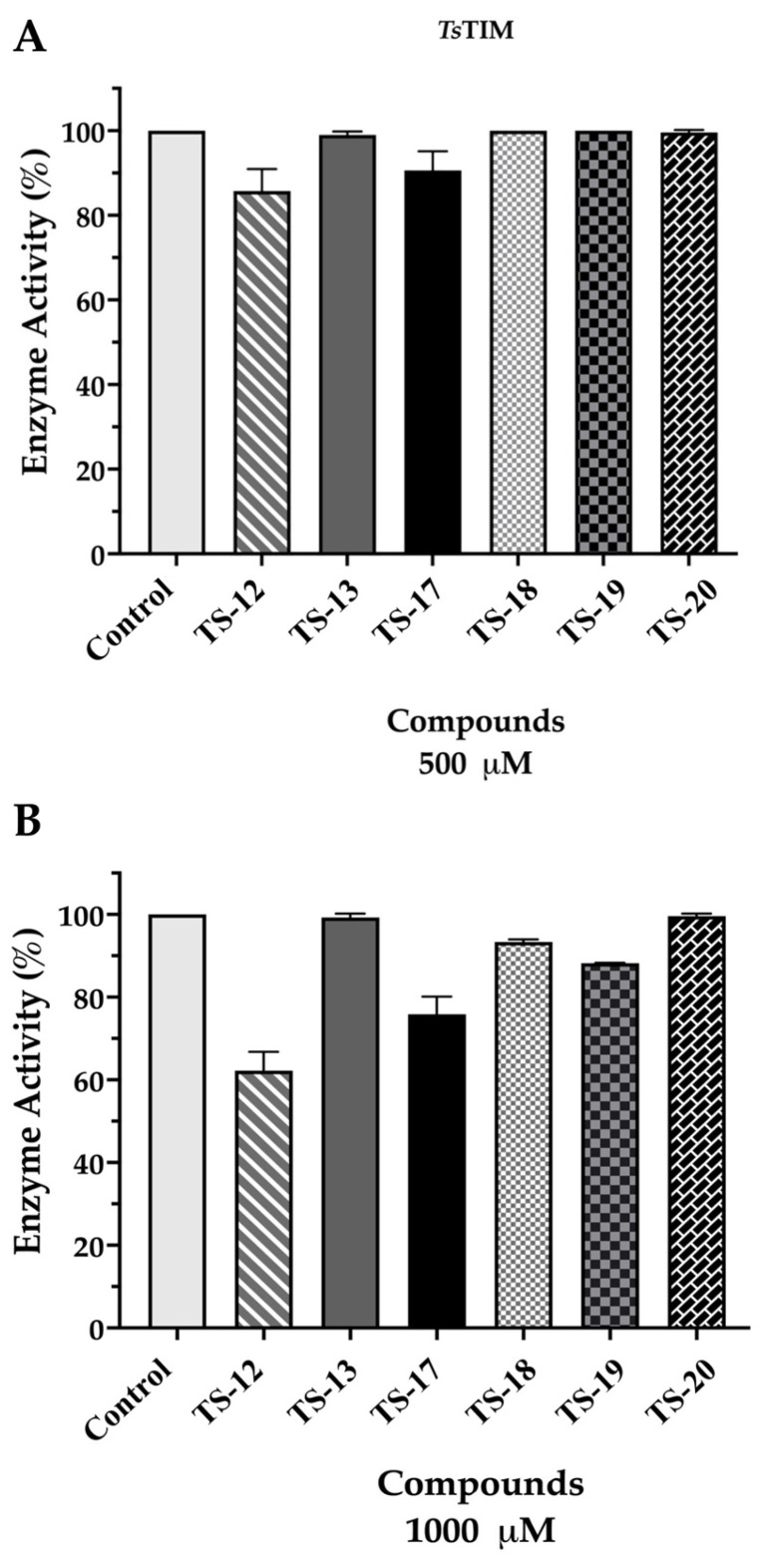
Enzyme activity of QNO derivatives against recombinant *Ts*TIM. (**A**) Results of enzyme activity percentage at 500 µM against *Ts*TIM in the GAP to DHAP direction. (**B**) Results of enzyme activity percentage at 1000 µM; TS-11 and TS-15 inhibited 38% and 36%, respectively.

Compounds with the best cysticidal activity and those with the highest predicted binding affinity against *Ts*TIM (TS-12, TS-13, TS-17, TS-18, TS-19, and TS-20) were evaluated through an in vitro enzymatic to determine their effect as *Ts*TIM inhibitors. Figure 4 summarizes the results.

## 3. Discussion

### 3.1. Structure–Activity Relationship Analysis

The results show that methyl esters of quinoxaline 1,4-di-*N*-oxide derivatives have no biological effects against *T. crassiceps,* except for the compound TS-06 with an EC_50_ value of 5.34 µM. TS-06 has a thiophene group at the two-position and a trifluoromethyl group at the three-position on the quinoxaline 1,4-di-*N*-oxide ring. Similarly, in the ethyl and n-propyl series, all derivatives were inactive (EC_50_ > 10 µM) except for the TS-12 (EC_50_ = 0.58 µM) and TS-15 (EC_50_ = 3.87 µM) compound derivatives that have thiophene and trifluoromethyl groups at the two- and three-position, respectively, thus emphasizing the benefit of both substituents. The comparison of TS-06, TS-12, and T-015 shows that the optimal elongation of the aliphatic chain corresponds to two carbons. Interestingly, all 7-isopropyl esters of quinoxaline-1,4-di-*N*-oxide derivatives (TS-17, TS-18, TS-19, and TS-20) have EC_50_ values under 3 µM, which shows that the addition of the isopropyl group is key in biological activity.

**Figure 5:**
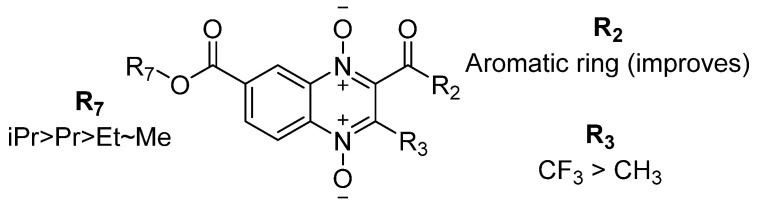
Summary of the beneficial groups at two-, three-, and seven-position on the quinoxaline-1,4-di-*N*-oxide ring regarding its antitaeniasis activity.

The comparison of the methyl (TS-02), ethyl (TS-08), and isopropyl (TS-18) ester derivatives with a phenyl ketone at the two-position and a methyl at the three-position showed a positive effect of the isopropyl group at the seven-position, as TS-18 is over 8-fold more active than both TS-02 and TS-08. Similarly, the comparison of derivatives TS-06, TS-12, TS-15, and TS-20 shows that the modification of the ester chain from methyl to ethyl enhances 9.2-times the antitaeniasis activity. However, an elongation into an n-propyl chain results in a 6.7-times decrease in activity, and the ramification of n-propyl into isopropyl ester permits a 4.8-times increase, suggesting the next order in the activity ethyl~isopropyl > n-propyl > methyl. Moreover, the comparison of quinoxaline 1,4-di-*N*-oxide derivatives with a methyl group versus a trifluoromethyl group at the three-position shows that trifluoromethyl improves the antitaeniasis activity. Finally, at the two-position, aromatic groups are important in the effects over other substitutions. Figure 5 summarizes these findings following SAR analysis.

### 3.2. Molecular Docking on TsTIM

The top five compounds (TS-02, TS-08, TS-13, TS-16, and TS-18) have BFE values ranging from −6.426 to −6.679 kcal/mol. The most frequent interactions (>60%) occur with three residues, Phe101, Leu107, and Lys67, each of which form part of the hydrophobic portion of the interface that has been related to the stability of the dimer and the activity of the enzyme; except for TS-18 (which has only two out of three) these five derivatives had interactions with all three of the relevant residues. TS-02, TS-08, TS-13, and TS-16 had interactions with four out of the eight residues that comprise the hydrophobic portion, one of which belongs to residues not shared in human homolog Ile100. TS-18 had interactions with three out of the eight residues that comprise the hydrophobic portion; still, it does not bear interaction with a residue that is not shared by human homologs. Two amino acid residues that may be regarded as key to the binding of most quinoxaline-1,4-di-*N*-oxide derivatives are Phe101 and Leu107, both of which are part of the interaction profile in 18 and 19 out of 20 derivatives (90 and 95%, respectively); the remaining interaction residues vary considerably depending on their substituents at the R2, R3, and R7 positions.

**Figure 6:**
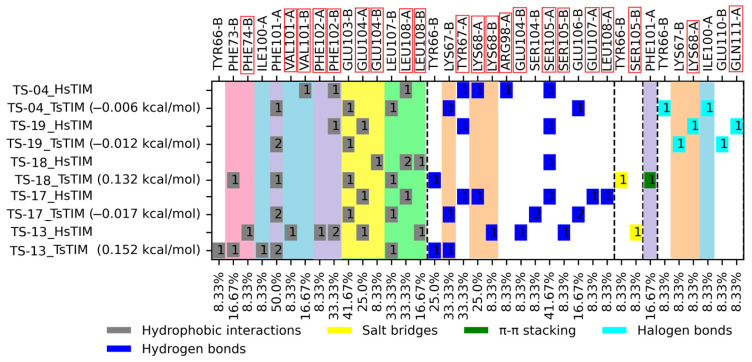
Interaction profile analysis for the top five compounds scored with the best BFE values; shared residues between parasite and human protein are shaded in the same color. *Hs*TIM residues are marked by red rectangles. The number within the box reflects the number of interactions of that type for the given residue.

Two quinoxaline derivatives (TS-13 and TS-18) had the highest predicted selectivity toward *Ts*TIM over *Hs*TIM (which bears a global residue identity of 59.29%), having better BFE toward *Ts*TIM over *Hs*TIM. Three additional quinoxaline derivatives (TS-04, TS-17, and TS-20) have a slight preference for *Hs*TIM; yet, having only a BFE value difference less than 0.02 kcal/mol favoring *Hs*TIM makes them worth exploring for their potential affinity on *Ts*TIM. Figure 6 below shows the positionally equivalent residues between *T. solium* and the human triosephosphate isomerase hydrophobic cavity as columns shaded in the same color.

**Figure 7:**
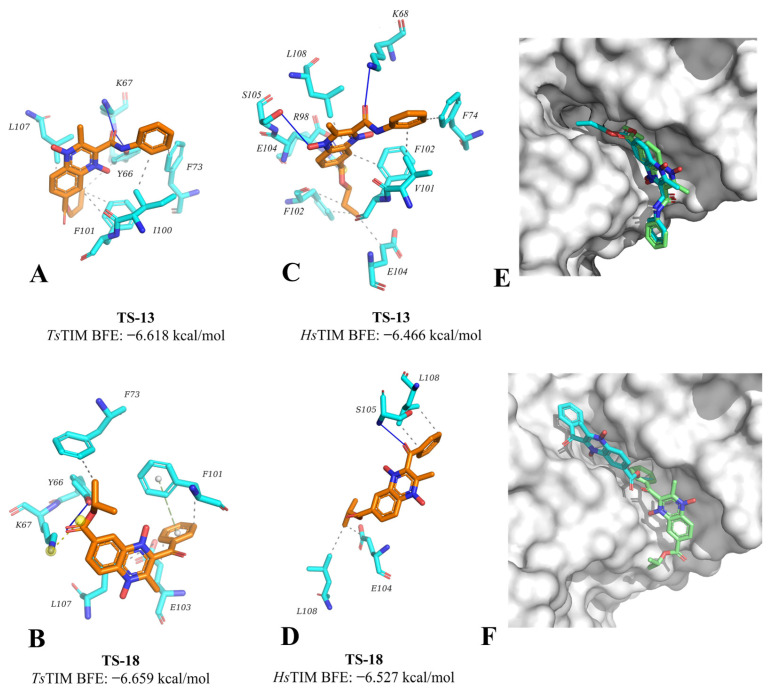
Docking pose for TS-12 and TS-16 on *Ts*TIM and *Hs*TIM, (**A**–**D**): individual poses; overlapped poses (green, *Ts*TIM; cyan, *Hs*TIM), (**E**): TS-12, (**F**): TS-16.

Further analyzing the interactions between the compounds with affinity potential on *Ts*TIM, it may be observed that three derivatives shared two or three interactions with important residues and are not predicted for *Hs*TIM (TS-04, TS-13, and TS-18). Considering both criteria, it is suggested that from these two compounds, TS-13 and TS-18 may be proposed to act with selective affinity, as they hold the most interactions with residues of interest on *Ts*TIM that are not predicted for *Hs*TIM. Figure 7 shows the interaction profile observed for the most promissory derivatives TS-13 and TS-18 on *Ts*TIM and *Hs*TIM, where it may be observed that the docking pose for TS-13 overlaps almost exactly for both proteins. The docking on *Hs*TIM is closer to the cavity wall, whereas for *Ts*TIM, it is centered between both walls; still, both interact with residues in both chains. On the other hand, the docking pose for TS-18 shows the derivative on *Ts*TIM binds clearly within the cavity, whereas for *Hs*TIM, it is slightly leaning toward the periphery of the cavity, suggesting that this derivative holds an important potential for selective inhibition of *Ts*TIM over *Hs*TIM. Altogether, this suggests that the cysticidal activity of TS-16 may be related to its affinity for *Ts*TIM. Still, further studies assessing the in vitro affinity for these compounds on *Hs*TIM are required to confirm their potential selectivity.

### 3.3. Enzymatic Activity Evaluation Against TsTIM

The results demonstrate that TS-12, TS-13, TS-17, TS-18, TS-19, and TS-20 derivatives have null inhibitory activity on *Ts*TIM at concentrations of 500 µM (Figure 4A). However, at the highest assayed concentration (1000 µM), an enzyme inhibition < 40% was determined for the compound TS-12 (Figure 4B). These results suggest that the biological activity for the most active esters of quinoxaline 1,4-di-*N*-oxide derivatives is not related to *Ts*TIM, as the required concentration to induce even a slight inhibition is over 100-fold higher than the concentrations assayed for the in vitro antitaeniasis activity.

## 4. Materials and Methods

### 4.1. Synthesis

The derivatives were synthesized and structurally elucidated according to the previous reports [26,34,35,36,37] (Supplementary Material).

### 4.2. Parasite Culture

The in vitro cysticidal activity assays were performed on *T. crassiceps* cysts (ORF strain). This model has been previously used for the screening of cysticidal drugs, as it bears an important similarity with *T. solium* cysts, as well as the ease to maintain cysts indefinitely in inbred mice [38,39]. Cysts were obtained from female BALB/c mice that were experimentally infected; both the infection and the obtention of cysts were carried out according to a procedure previously described. Briefly, after 3 months of infection, mice were euthanized by cervical dislocation, and metacestodes were removed from the peritoneal cavity and washed several times with sterile 0.9% saline solution. For assays, parasites measuring 2–3 mm, which had undergone no budding, possessed a translucent membrane, and exhibited intact bladder surface were used [40]. All the studies were approved by the Institutional Committee for Handling and Animal Care of the Instituto Nacional de Neurología y Neurocirugía (Register 146/21) and were carried out according to Mexican Guidance (NOM-062-ZOO-1999).

### 4.3. Drugs and Reagents

The study used albendazole sulfoxide (ABZSO, the active metabolite of ABZ) as a positive control, which was purchased from Sigma-Aldrich Chemical Co. (St. Louis, MO, USA). Analytical-grade dimethyl sulfoxide (DMSO) and ethanol were purchased from J.T. Baker (Xalostoc, México). Dulbecco’s modified Eagle’s medium-high glucose (DMEM) was used for the cysticidal evaluation and was purchased from Sigma-Aldrich Co. (St. Louis MO, USA). DMEM was supplemented with 10% fetal calf serum (Biowest- Mayimex, Veracruz, México), 2 mM L-glutamine, 8 mg/dL of gentamicin sulfate, and 200,000 IU/dL of penicillin G sodium (Gibco, Gran Island, NY, USA).

### 4.4. In Vitro Cysticidal Activity Against T. crassiceps

A stock solution of each quinoxaline 1,4-di-*N*-oxide in DMSO was prepared at 1 mM for the evaluation. Then, a 1:10 dilution was carried out for 100 µL of the stock solution by diluting it using water to a final volume of 1 mL to obtain a work solution of 100 µM. Different aliquots were taken from the solution of 100 µM to prepare the solutions of quinoxaline 1,4-di-*N*-oxide derivatives in DMEM in the concentration range from 0.05 to 20 µM. A stock solution of ABZSO (positive control) was prepared in DMSO at 1 mM, and this solution was serially diluted to prepare a solution in DMEM in the same range of concentration as quinoxaline 1,4-di-*N*-oxide derivatives. DMSO 0.1% in DMEM was prepared as the negative control.

Twenty-four-well cell culture flat-bottom microplates (Corning, Kennebunk, ME, USA) were used for the assay. Each well was filled with a final volume of 2 mL of DMEM containing each compound or the control. Ten cysts were carefully deposited into each well and then were incubated at 37 °C with 5% CO_2_ atmosphere and 98% relative humidity for 11 days. The medium was replaced every two days, the cysts were monitored daily, and the mortality was registered using an inverted light microscope ICM 405 (Carl Zeiss Inc., Pleasanton, CA, USA). The loss of vesicular fluid, paralysis of the membrane, and collapse of parasites were the criteria to assess mortality [41], and a Trypan Blue Exclusion test was conducted to confirm the mortality at day 12 [42]. All treatments were performed in triplicate, and two different experiments were conducted. The concentration–response curves were fitted, and the effective concentration required to kill 50% of the cysts (EC_50_) and 95% confidence limits were determined using non-linear regression and the GraphPad Prism software (V. 8.0.2 for Windows, MA, USA).

### 4.5. Molecular Docking

Molecular docking analysis evaluated the possible interaction of the quinoxaline-7-carboxylate 1,4-di-*N*-oxide derivatives against *Ts*TIM. The quinoxaline structures were drawn using the Marvin Sketch software 21.13 [43], 3D-energy minimized using MMFF94 forcefield, and Gaisterger partial charges were added with Open Babel (v 3.1.1) [44] and saved in pdb format. The crystal structure for the protein receptor of *Ts*TIM was retrieved from the Protein Data Bank (PDB) with the four-letter access code 6OOG [45]. The initial preparation of the protein receptor for molecular docking included removing water molecules, any co-crystalized ligands, and/or ions from the protein structure. It was then that an energy-minimization step was performed using the Yasara Minimization Server (YASARA forcefield) (accessed January 2024) and further prepared with Chimera 1.15 by adding Gaisteiger partial charges [46] before docking simulations. All docking simulations were performed with AutoDock VINA 1.2.5 [47,48].

Previous findings of quinoxaline-1,4-di-*N*-oxide derivatives as TIM inhibitors suggest that these bind to the interface portion of the TIM enzyme [33], further confirmed by DoGSite3 analysis using ProteinsPlus server [49,50,51]. The molecular docking calculations were performed at the interface coordinate space (X = 9.302, Y = −7.492, and Z = 32.315) using a grid box measuring 24 Å in each of the XYZ dimensions, considering a grid spacing of 1 Å. Two criteria were considered for the analysis of the results: the BFE value and the interaction profile. Initially, the highest calculated BFE for each protein–ligand complex was considered. These promissory poses were then analyzed to calculate their molecular interactions between *Ts*TIM and the quinoxaline 1,4-di-*N*-oxide derivative utilizing the protein–ligand interaction profiler (PLIP) software version 2.2.2 [52].

### 4.6. Expression and Purification of Recombinant TsTIM

Recombinant *Ts*TIM was overexpressed and purified. The gene encoding the recombinant enzyme was in pRSET (Invitrogen, Waltham, MA, USA) and was expressed in *Escherichia coli* BL21 Codon Plus (DE3)-RIL strain. The pRSET includes a sequence that encodes an N-terminal tag of six histidine residues that allows the purification of recombinant *Ts*TIM by affinity chromatography. Bacteria transformed with the plasmid for WT *Ts*TIM were grown in an LB medium supplemented with 0.1 mg/mL of ampicillin and 0.050 mg/mL of chloramphenicol and incubated at 37 °C. Once the culture reached Abs_600nm_ = 0.8, they were induced by using 1-mM IPTG and further incubated overnight at 30 °C with constant shaking at 180 rpm. After induction and overnight growth, the bacteria were collected by centrifugation (4010× *g*, 15 min) and suspended in 40 mL of lysis buffer (100 mM Triethanolamine, pH 8.0, 50 mM NaCl, and 1 mM DTT, 0.5 mM PMSF). Sonication was used to disrupt the bacterial suspension; it was then centrifuged for 1 h at 7690× *g* at 4 °C. The enzyme was purified by immobilized metal affinity chromatography (IMAC) using a Profinity Ni^2+^-charged resin previously equilibrated with lysis buffer. The soluble protein fraction was then mixed with the equilibrated Ni^2+^-charged resin and further incubated at 4 °C with shaking for 30 min. The column was washed with the same buffer (10 column volumes) to remove proteins without the His-tag sequence. *Ts*TIM was eluted with lysis buffer containing 250 mM imidazole and adjusted to pH 8.0. The purified protein was concentrated to a volume of 1 mL using Centricon filter units (Millipore, Billerica, MA, USA). Protein concentration was determined spectrophotometrically at 280 nm using an estimated molar extinction coefficient of 36,440 M^−1^ cm^−1^ for WT *Ts*TIM [53].

### 4.7. Enzyme Inactivation Assays of Recombinant TsTIM

The quinoxaline-1,4-di-*N*–oxide derivatives TS-12, TS-13, TS-17, TS-18, TS-18, and TS-20 were freshly dissolved in DMSO before use. The *Ts*TIM inactivation assays were performed at 0.2 mg/mL protein concentrations in TE buffer (100 mM Triethanolamine, pH 7.4, 10 mM EDTA) for 2 h at 37 °C. Samples from these incubations were collected and assayed for enzyme activity. The enzymatic activity of the purified recombinant *Ts*TIM was assessed by measuring the conversion of glyceraldehyde 3-phosphate (GAP) to dihydroxyacetone phosphate (DHAP). Briefly, this conversion reaction was monitored spectrophotometrically by tracking NADH oxidation at 340 nm using α-GDH as the coupling enzyme (Spectrophotometer Cary 50, Varian Inc., Sydney, Australia). The samples incubated without any derivative were set as 100% enzyme activity and compared with experimental conditions. The reactions were initiated upon the addition of 5 ng/mL *Ts*TIM to the reaction mixture [53]. The high concentrations of derivatives used in these assays were required to achieve conditions approaching equimolarity between the derivatives and recombinant *Ts*TIM. It is important to note that while recombinant *Ts*TIM was present at a high concentration, cellular *Ts*TIM may be present at a significantly lower concentration. Additionally, high concentrations of derivatives were necessary due to the short incubation times employed in these experiments.

## 5. Conclusions

In this study, the biological evaluation of quinoxaline-1,4-di-*N*-oxide against *T. crassiceps* demonstrates that these compounds are effective antitaeniasis agents (TS-12, TS-19, and TS-20) with EC_50_ values similar to or equal to the reference drug, albendazole. The most beneficial substitutions are at the two-position, a thienyl group; at the three-position, a trifluoromethyl group; and at the seven-position, an isopropyl group. Although the molecular docking analysis shows that some compounds have the highest potential to behave as selective *Ts*TIM inhibitors at the enzyme interface, the enzymatic assays have shown a low effect on *Ts*TIM; therefore, it is necessary to explore their mode of action. To our knowledge, this is the first report that encourages the development of new antitaeniasis agents using the quinoxaline 1,4-di-*N*-oxide scaffold.

## Figures and Tables

**Table 1:** Antitaeniasis activity of esters of quinoxaline-1,4-di-*N*-oxide derivatives against *T. crassiceps*.

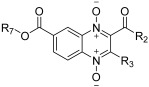					
Code	R_7_	R_2_	R_3_	EC_50_ (µM)	SD
TS-01	CH_3_	CH_3_CH_2_O	CH_3_	>10	
TS-02	CH_3_	C_6_H_5_	CH_3_	>10	
TS-03	CH_3_	NHC_6_H_5_	CH_3_	>10	
TS-04	CH_3_	CH_3_CH_2_	CF_3_	>10	
TS-05	CH_3_	CH(CH_3_)_2_	CF_3_	>10	
TS-06	CH_3_	C_4_H_3_S	CF_3_	5.34	1.06–2.11
TS-07	CH_3_CH_2_	CH_3_	CH_3_	>10	
TS-08	CH_3_CH_2_	C_6_H_5_	CH_3_	>10	
TS-09	CH_3_CH_2_	CH_3_	CF_3_	>10	
TS-10	CH_3_CH_2_	CH_3_CH_2_	CF_3_	>10	
TS-11	CH_3_CH_2_	CH(CH_3_)_2_	CF_3_	>10	
TS-12	CH_3_CH_2_	C_4_H_3_S	CF_3_	0.58	0.24–1.46
TS-13	CH_3_CH_2_CH_2_	NHC_6_H_5_	CH_3_	>10	
TS-14	CH_3_CH_2_CH_2_	C_4_H_3_O	CF_3_	>10	
TS-15	CH_3_CH_2_CH_2_	C_4_H_3_S	CF_3_	3.87	0.76–1.47
TS-16	CH_3_CH_2_CH_2_	C_6_H_5_	CF_3_	>10	
TS-17	(CH_3_)_2_CH	NH_2_	CH_3_	2.69	1.54–5.24
TS-18	(CH_3_)_2_CH	C_6_H_5_	CH_3_	1.75	1.04–3.25
TS-19	(CH_3_)_2_CH	CF_2_CF_3_	CF_3_	1.02	0.50–2.62
TS-20	(CH_3_)_2_CH	C_4_H_3_S	CF_3_	0.80	0.42–1.85
ABZSO				0.68	0.39–1.85

ABZSO: Albendazole sulfoxide.

## Data Availability

The data presented here, including synthetic methodology, can be openly shared upon request to the corresponding author: giriveras@ipn.mx.

## Supplementary Materials

The following supporting information can be downloaded at: https://www.mdpi.com/article/10.3390/ph18030406/s1. Methodology for the synthesis and structural elucidation of TS-1 to TS-18 derivatives.

## Abbreviations

The following abbreviations are used in this manuscript:

α-GDH	α-Glycerophosphate Dehydrogenase
ABZSO	Albendazole sulfoxide
BFE	Binding free energy
CNS	Central nervous system
DHAP	Dihydroxyacetone-3-phosphate
DMEM	Dulbecco’s modified Eagle’s medium
DMSO	Dimethyl sulfoxide
DTT	Dithiothreitol
EC_50_	Half-maximal effective concentration
EDTA	Ethylenediaminetetraacetic acid
GAP	Glyceraldehyde-3-phosphate
*Hs*TIM	*Homo sapiens* triosephosphate isomerase
IMAC	Immobilized metal affinity chromatography
IPTG	Isopropyl β-D-1-thiogalactopyranoside
LB medium	Lysogeny broth medium
µM	Micromolar
NADH	Nicotinamide adenine dinucleotide
PLIP	Protein–ligand interaction profiler
PMSF	Phenylmethylsulfonyl fluoride
QNO	Quinoxaline-1,4-di-*N*-oxide
SAR	Structure–activity relationship
*T. crassiceps*	*Taenia crassiceps*
*T. solium*	*Taenia solium*
TE buffer	Tris EDTA
TIM	Triosephosphate isomerase
*Ts*TIM	*Taenia solium* triosephosphate isomerase
WHO	World Health Organization
