# Supplementary material for: In Vitro Evaluation of Esters of Quinoxaline-1,4-di-N-oxide Derivatives as New Antitaeniasis Agents and Their Inhibitory Activity Against Triosephosphate Isomerase
Source: Pharmaceuticals (Basel). 2025 Mar 13;18(3):406. doi: 10.3390/ph18030406 (PMC11944476; doi:10.3390/ph18030406)
Supplement: Supplementary file 1 [file pharmaceuticals-18-00406-s001.zip › pharmaceuticals-3456887-supplementary.pdf]

In Vitro Evaluation of Esters of Quinoxaline-1,4-di-*N*-oxide Derivatives as New Antitaeniasis Agents and Their Inhibitory Activity Against Triosephosphate Isomerase

Francisca Palomares-Alonso [1,†], Alonzo González-González [2,†], Alma D. Paz-González [2], Eyra Ortiz-Pérez [2], Ana Verónica Martinez-Vazquez [2], Itzhel García-Torres [3], Gabriel López-Velázquez [3], Helgi Jung-Cook [4] and Gildardo Rivera [2,*]

1 Laboratorio Para el Estudio de la Neuro-Inflamación, Instituto Nacional de Neurología y Neurocirugía, Ciudad de México 14269, Mexico; ingpalomares@yahoo.com.mx

2 Laboratorio de Biotecnología Farmacéutica, Centro de Biotecnología Genómica, Instituto Politécnico
Nacional, Reynosa 88710, Mexico; al.gonzalez.gonzalez88@gmail.com (A.G.-G.); apazg@ipn.mx (A.D.P.-G.); eortizp@ipn.mx (E.O.-P.); avmartinez@ipn.mx (A.V.M.-V.)

3 Laboratorio de Biomoléculas y Salud Infantil, Instituto Nacional de Pediatría, México City 04530, Mexico; garciaitzhel@gmail.com (I.G.-T.); glv_1999@ciencias.unam.mx (G.L.-V.)

4 Facultad de Química, Departamento de Farmacia, Universidad Nacional Autónoma de México,
Ciudad de México 04510, Mexico; helgi@unam.mx

* Correspondence: giriveras@ipn.mx

† These authors contributed equally to this work.

Supplementary Material

Synthesis of TS-1 to TS-18 series

The Beirut reaction was the general procedure used for methyl, ethyl, propyl, and isopropyl quinoxaline-7-carboxylate 1,4-di-N-oxide derivatives synthesis, as described in previous reports (Gómez-Caro *et al.* 2011; Duque-Montano *et al.* 2013, Villalobos-Rocha *et al.* 2014, Chacon-Vargas *et al.* 2017, Palos *et al.* 2018). Quinoxaline-7-carboxylate 1,4-di-N-oxide derivatives synthesis was achieved by the reaction of the corresponding diketone derivative (10.6 mmol) with the appropriate benzofuroxan (2.4 mmol) in dry chloroform (35 ml). Triethylamine (TEA) was added (1 ml), and the reaction mixture was stirred at room temperature for 3–7 days. After evaporation to dryness at low pressure, crude solid or brown oil was obtained. This was then precipitated and washed by adding diethyl ether, affording the target compound. The residue was purified by column chromatography on silica gel, when necessary, using dichloromethane/methanol (95:5). All quinoxaline-7-carboxylate 1,4-di-*N*-oxide derivatives were characterized by infrared (IR), proton nuclear magnetic resonance (^{1}H NMR) spectroscopy, and elemental analysis, as previously reported.

NMR

TS-01. 3-(ethoxycarbonyl)-6-(methoxycarbonyl)-2-methylquinoxaline 1,4-dioxide: ^{1}H NMR (400 MHz, DMSO-d_6) δ-ppm: 1.32(t, 3H, CH_3CH_2O) 2.45 (s, 3H, CH_3), 3.97 (s, 3H, CH_3O), 4.52 (c, 2H, CH_3CH_2O), 8.39 (d, J= 1.80 and 8.97 Hz, 1H, H_6), 8.56 (d, J= 1.72 Hz, 1H, H_5), 8.86 (m, 1H, H_8).

TS-02. 3-benzoyl-6-(methoxycarbonyl)-2-methylquinoxaline 1,4-dioxide: ^{1}H-NMR (400 MHz, DMSO-d_6) δ-ppm: 2.32 (s, 3H, CH_3), 3.99 (s, 3H, CH_3O), 7.59 (t, J= 7.67 Hz, 2H, H_3 and H_5, C_6H_5), 7.78 (t, J= 7.33 Hz, 1H, H4, C_6H_5), 8.08 (d, J= 7.51 Hz, 2H, H_2 and H_6, C_6H_5), 8.37 (d, J= 1.17 Hz, 1H, $H_{6,}$), 8.49 (d, 1H, H_5), 8.96 (m, 1H, H_8).

TS-03. 6-(methoxycarbonyl)-2-methyl-3-(phenylcarbamoyl)quinoxaline 1,4-dioxide: ^{1}H NMR (400 MHz, DMSO-d_6) δ-ppm: 2.52 (s, 3H, CH_3), 3.99(s, 3H, CH_3O), 7.2 (t, J= 7.38 Hz, 1H, NHC_6H_5), 7.42 (t, J= 7.78 Hz, 2H, H_3 and H_5, NHC_6H_5), 7.67 (d, J= 7.92 Hz, 2H, H_2 and H_6, NHC_6H_5), 8.41 (d, J= 8.84 Hz, 1H, H_6), 8.62 (d, J= 8.93 Hz, 1H, H_5), 9.0 (s, 1H, H_8), 11.01 (s, 1H, NH).

TS-04. 6-(methoxycarbonyl)-3-propionyl-2-(trifluoromethyl)quinoxaline 1,4-dioxide: ^{1}H NMR (400 MHz, DMSO-d_6) δ ppm**:** 1.34 (t, J= 6.95 Hz, 3H, $COCH_2CH_3$), 2.99 (s, 2H, $COCH_2CH_3$), 4.07 (s, 3H, CH_3OOC), 8.57 (d, J= 8.88 Hz, 1H, H5), 8.65 (d, J= 9.01 Hz, 1H, H6), 9.26 (s, 1H, H8).

TS-05. 3-isobutyryl-6-(methoxycarbonyl)-2-(trifluoromethyl)quinoxaline 1,4-dioxide: ^{1}H NMR (400 MHz, DMSO-d_6) δ ppm: 1.27 (s, 6H, $CH(CH_3)_2$), 3.17 (q, J_1 = 14.09 Hz, J_2 = 7.05 Hz, 1H, $CH(CH_3)_2$), 4.07 (s, 3H, CH_3OOC), 8.57 (d, J = 8.98 Hz, 1H, H5), 8.64 (d, J = 8.95 Hz, 1H, H6), 9.27 (s, 1H, H8).

TS-06. 3-acetyl-6-(ethoxycarbonyl)-2-methylquinoxaline 1,4-dioxide: ^{1}H NMR (400 MHz, DMSO-d_6) δ ppm: 1.40 (t, J= 7.10 Hz, 3H, CH_3CH_2OOC), 2.38 (s, 3H, CH_3), 2.66 (s, 3H, $COCH_3$), 4.4 (q, J= 7.09 Hz, 2H, CH_3CH_2OOC), 8.37 (d, J= 8.93 Hz, 1H, H5), 8.5 (d, J= 8.94 Hz, 1H, H6), 8.92 (s, 1H, H8).

TS-07. 3-benzoyl-6-(ethoxycarbonyl)-2-methylquinoxaline 1,4-dioxide: ^{1}H NMR (400 MHz, DMSO-d_6) δ-ppm: 1.4 (t, J= 7.11 Hz, 3H, CH_3CH_2O), 2.32 (s, 3H, CH_3), 4.5 (c, J= 7.10 Hz, 2H, CH_3CH_2O), 7.6 (t, J= 7.79 Hz, 2H, H_3 and H_5, C_6H_5), 7.8 (t, J= 7.43 Hz, 1H, H_4, C_6H_5), 8.1 (d, J= 7.35 Hz, 2H, H_2 and H_6, C_6H_5), 8.4 (d, J= 1.72 Hz, 1H, H_6), 8.5 (s, 1H, H_5), 8.92 (m, 1H, H_8).

TS-08. 3-acetyl-6-(ethoxycarbonyl)-2-(trifluoromethyl)quinoxaline 1,4-dioxide: ^{1}H NMR (400 MHz, DMSO-d_6) ppm: 1.40 (t, J = 7.11 Hz, 3H, CH_3CH_2OOC), 2.62 (s, 3H, $COCH_3$), 4.45 (q, J_1 = 7.12 Hz, J_2 = 7.13 Hz, 2H, CH_3CH_2OOC), 8.52 (d, J = 8.95 Hz, 1H, H5), 8.58 (d, J = 8.96 Hz, 1H, H6), 8.92 (s, 1H, H8).

TS-09. 6-(ethoxycarbonyl)-3-propionyl-2-(trifluoromethyl)quinoxaline 1,4-dioxide: ^{1}H NMR (400 MHz, DMSO-d_6) ppm: 1.35 (t, 3H, $COCH_2CH_3$), 1.39 (t, 3H, CH_3CH_2OOC), 4.45 (q, J_1 = 6.99 Hz, J_2 = 14.10 Hz, 2H, $COCH_2CH_3$), 4.5 (q, J_1 = 7.05 Hz, J_2 = 14.17 Hz, 2H, CH_3CH_2OOC), 8.50 (d, J = 8.96 Hz, 1H, H5), 8.56 (d, J = 8.94 Hz, 1H, H6), 8.88 (s, 1H, H8).

TS-10. 6-(ethoxycarbonyl)-3-isobutyryl-2-(trifluoromethyl)quinoxaline 1,4-dioxide: ^{1}H NMR (400 MHz, DMSO-d_6) δ ppm: 1.21 (s, 6H, $CH(CH_3)_2$), 1.40 (t, J= 7.11 Hz, 3H, CH_3CH_2OOC), 2.42 (q, J_1= 7.16 Hz, 1H, $CH(CH_3)_2$), 4.45 (q, J= 7.09, 2H, CH_3CH_2OOC), 8.50 (d, J= 8.95 Hz, 1H, H5), 8.57 (d, J= 8.93 Hz, 1H, H6), 8.91 (s, 1H, H8).

TS-11. 6-(ethoxycarbonyl)-3-(thiophene-2-carbonyl)-2-(trifluoromethyl)quinoxaline 1,4-dioxide: ^{1}H NMR (400 MHz, DMSO-d_6) δ-ppm: 1.4 (t, J= 7.11 Hz, 3H, CH_3CH_2O); 4.5 (q, J= 7.07 Hz, 2H, CH_3CH_2O), 7.30-7.33 (m, 1H, H_4, C_4H_3S), 8.2 (dd, J= 3.83 and 0.76 Hz, H_5, C_4H_3S), 8.3 (dd, J= 4.83 and 0.74 Hz, H_3, C_4H_3S), 8.50-8.57 (m, 2H, H_5 and H_6), 8.96 (m, 1H, H_8).

TS-12. 2-methyl-3-(phenylcarbamoyl)-6-(propoxycarbonyl)quinoxaline 1,4-dioxide: ^{1}H-NMR (400 MHz, DMSO-d_6) δ-ppm: 1.02 (t, 3H, $CH_3(CH_2)_2O$), 1.8 (q, J_1 = 6.97 Hz, J_2 = 13.90 Hz, 2H, $CH_3(CH_2)_2O$), 2.51 (s, 1H, CH_3), 4.36 (t, J = 6.29 Hz, $CH_3(CH_2)_2O$), 7.20 (t, J = 7.35 Hz, 2H, H3 and H5, NHC_6H_5), 7.42 (t, J = 7.47 Hz, 1H, H4, NHC_6H_5), 7.67 (d, J = 7.83 Hz, 2H, H2 and H6, NHC_6H_5)) 8.4 (d, J = 8.88 Hz, 1H, H5), 8.6 (d, J = 8.82 Hz, 1H, H6), 9.0 (s, 1H, H8), 11.02 (s, 1H, NH).

TS-13. 3-(furan-2-carbonyl)-6-(propoxycarbonyl)-2-(trifluoromethyl)quinoxaline 1,4-dioxide:

TS-14. 3-benzoyl-6-(propoxycarbonyl)-2-(trifluoromethyl)quinoxaline 1,4-dioxide

TS-15. 3-carbamoyl-6-(isopropoxycarbonyl)-2-methylquinoxaline 1,4-dioxide: ^{1}H-NMR (400 MHz, DMSO-d_6) δ-ppm: 1.38 (s, 6H, $(CH_3)_2CH$), 2.48 (s, 3H, CH_3), 5.20-2.27 (m, $(CH_3)_2$-CH-), 8.20 (s, 2H, $CONH_2$), 8.38 (d, J = 9.17 Hz, 1H, H5), 8.58 (d, J = 8.77 Hz, 1H, H6), 8.90 (s, 1H, H8).

TS-16. 3-benzoyl-6-(isopropoxycarbonyl)-2-methylquinoxaline 1,4-dioxide: ^{1}H-NMR (400 MHz, DMSO-d_6) δ-ppm: 1.40 (s, 6H, $(CH_3)_2CH$), 2.30 (s, 3H, CH_3), 5.3 (q, J_1 = 6.23 Hz, J_2 = 12.30 Hz, $(CH_3)_2$-CH-), 7.6 (t, J = 7.84 Hz, 2H, H3 and H5, C_6H_5), 7.8 (t, J = 7.95 Hz, 1H, H4, C_6H_5), 8.1 (d, J = 8.28 Hz, 2H, H2 and H6, C_6H_5), 8.4 (d, J = 8.4 Hz, 1H, H5), 8.5 (d, J = 8.95 Hz, 1H, H6), 8.97 (s, 1H, H8).

TS-17. 6-(isopropoxycarbonyl)-3-(2,2,3,3,3-pentafluoropropanoyl)-2-(trifluoromethyl)quinoxaline 1,4-dioxide: ^{1}H-NMR (400 MHz, DMSO-d_6) δ-ppm: 1.38 (s, 6H, $(CH_3)_2CH$), 5.22–5.26 (m, $(CH_3)_2$-CH-), 8.51 (d, J = 9.5 Hz, 1H, H5), 8.62 (d, J = 9.4 Hz, 1H, H6), 8.9 (s, 1H, H8).

TS-18. 6-(isopropoxycarbonyl)-3-(thiophene-2-carbonyl)-2-(trifluoromethyl)quinoxaline 1,4-dioxide: ^{1}H-NMR (400 MHz, DMSO-d_6) δ-ppm: 1.40 (m, 6H, $(CH_3)_2CH$), 5.24–5.29 (m, $(CH_3)_2$-CH-), 7.31 (s, 1H, H4, C_4H_3S), 8.24 (s, H5, C_4H_3S), 8.30 (s, H3, C_4H_3S), 8.51 (s, 1H, H5), 8.54 (s, Hz, 1H, H6), 8.94 (s, 1H, H8).
